# Variation in the Sodium-Dependent Vitamin C Transporter 2 Gene Is Associated with Risk of Acute Coronary Syndrome among Women

**DOI:** 10.1371/journal.pone.0070421

**Published:** 2013-08-21

**Authors:** Christine Dalgård, Lene Christiansen, Ulla Vogel, Claus Dethlefsen, Anne Tjønneland, Kim Overvad

**Affiliations:** 1 Department of Environmental Medicine, Institute of Public Health, University of Southern Denmark, Odense, Denmark; 2 Department. of Epidemiology, Institute of Public Health, University of Southern Denmark, Odense, Denmark; 3 National Research Centre for the Working Environment, Copenhagen, Denmark; 4 Department of Cardiology, Center for Cardiovascular Research, Aalborg Hospital, Aarhus University Hospital, Aalborg, Denmark; 5 Danish Cancer Society Research Center, Copenhagen, Denmark; 6 Section for Epidemiology, Department of Public Health, Aarhus University, Aarhus, Denmark; Scuola Superiore Sant'Anna, Italy

## Abstract

**Background:**

Vitamin C is associated with a lower risk of coronary heart disease possibly due to its anti-oxidative effects, beneficial effects on endothelial function and importance in collagen synthesis. The sodium-dependent vitamin C transporter 2 is responsible for the transport of vitamin C into various cells and malfunction of this protein leads to reduced vitamin C in tissue, including the arterial wall. We tested the hypothesis that candidate variations rs6139591 and rs1776964 in the gene coding for sodium-dependent vitamin C transporter 2 are associated with development of acute coronary syndrome.

**Design:**

In the Danish Diet, Cancer and Health cohort study, we performed a case-cohort study among 57,053 subjects aged 50–64 years.

**Results:**

During a mean follow-up period of 6.4 years, we identified 936 cases and randomly selected a sub-cohort (n = 1,580) with full information on genotypes and covariates. Using Cox proportional hazard models, we found that women with the rs6139591 TT genotype and a lower than median dietary vitamin C intake had a higher risk of acute coronary syndrome compared with those with the CC genotype (adjusted HR 5.39, 95% confidence interval, 2.01–14.50). We also observed a not as strong but positive although inconsistent association for women at a higher than median intake of vitamin C rich food. For the rs1776964 polymorphism, we found a higher risk (adjusted HR 3.45, 95% CI, 1.16–10.28) among TT-homozygous women with higher than median vitamin C intake compared with the CC genotype and low vitamin C intake. Among men, weaker and non-significant associations were observed for both polymorphisms.

**Conclusion:**

Genetic variation in the sodium-dependent vitamin C transporter 2 is associated with risk of incident acute coronary syndrome in women. The genotype effects may not be fully compensated by a higher intake of vitamin C rich food.

## Introduction

Acute coronary syndrome (ACS) is still among the leading causes of mortality in Denmark as well as internationally [1] despite recent declines in mortality rates. It is estimated that ca. 50% of the decrease is explained by reductions in the well-known risk factors, i.e. blood cholesterol level, blood pressure, and smoking [2]. A recent report suggests that ca. 90% of the population attributable risk can be related to 9 specific risk factors including low intake of fruit and vegetables [3] that have consistently been associated with risk of cardiovascular disease [4]. Though the pathogenic mechanisms of low fruit and vegetable intake are far from being completely understood, specific nutrients are likely to play a role.

One of the main cardioprotective factors in fruits and vegetables may be vitamin C and numerous mechanisms have already been identified including anti-oxidative effects and beneficial effects on endothelial function. Vitamin C is also important in collagen synthesis; hence vitamin C deficiency may reduce the collagen content of the atherosclerotic plaques leading to vulnerable plaques more likely to rupture [5].

Sodium dependent vitamin C transporters (SVCT) 1 and 2 function to keep vitamin C homeostasis, but while the SVCT1 is primarily expressed in intestinal and renal tissue controlling vitamin C uptake and excretion the SVCT2 is expressed in metabolically highly active tissue [6] [7] securing intracellular ascorbate accumulation against its concentration gradient in several tissues, including aorta [8]. This intracellular ascorbic acid concentration is a co-determinant of the collagen synthesis in the arterial wall as well as the plaque cap. Furthermore, the intracellular vitamin C concentration may reduce endothelial dysfunction and inflammation, which are hallmarks of the vulnerable plaque [9]. The SVCT2 protein is encoded by *SLC23A2* that maps to human chromosome 20p12. In an animal model, the gene was essential for survival, as SVCT2 knock-out mice died within minutes after birth [10]. In humans, various single nucleotide polymorphisms (SNPs) were associated with various diseases and conditions, including several types of cancers [11]–[14] and preterm birth [15]. Furthermore, Cahill and colleagues genotyped SNPs at the *SLC23A2* locus and found that two SNPs (rs6139591 and rs2681116) modified the association correlation between vitamin C intake and circulating ascorbic acid concentration [16]. However, so far no studies have determined whether SNPs in *SLC23A2* affect cardiovascular risk, hence using two single nucleotide polymorphisms that have previously been associated with spontaneous rupture of collagen membranes in Caucasian women, i.e. rs6139591 and rs1776964 in the *SLC23A2* locus as genetic markers, we determined the possible association between SVCT2 variants and incident ACS in a prospective study. In addition, we hypothesized a modifying effect of a low dietary intake of vitamin C.

## Materials and Methods

### Ethic statement

All subjects gave written informed consent and the study was approved by the Ethical Committees on Human Studies for the Copenhagen and Aarhus municipalities (H-KF-01-345/93) and by the Danish Data Protection Agency.

### Study population

Between 1993 and 1997, 160,725 individuals aged 50–64 years were invited to participate in a Danish prospective follow-up study, the Diet, Cancer and Health study. Eligible participants were born in Denmark and without records of cancer in the Danish Cancer registry and lived in the urban areas of Aarhus and Copenhagen. In total, 27,178 men (33.6% of total number of eligible) and 29,875 women (37.5% of total number of eligible) participated.

The participants underwent a physical examination including measures of height (to the nearest half centimetre) and body weight (to the nearest 100 grams). Body mass index (BMI) was calculated as body weight divided by height in meters squared. Blood samples were obtained at baseline and plasma, serum, lymphocytes and erythrocytes were isolated. Samples were stored at −150°C. Questionnaires included questions regarding diet, lifestyle, smoking habits, alcohol habits, medical treatment and other socioeconomic characteristics and environmental exposures, including use of dietary supplements.

A case-cohort study was designed using incident acute coronary syndrome cases (ACS) including myocardial infarction, unstable angina pectoris and sudden death as the outcome from 1994 through to December 31, 2003. Information on the endpoint was obtained by linkage with the Danish central registries via the unique identification number assigned at birth to all Danish citizens [17]. For all possible cases identified in the Danish National Patient Registry medical records were retrieved from hospitals for participants who were registered with a first-time discharge diagnosis of ACS (ICD-8 codes 410–410.99, 427.27 and ICD-10 codes I20.0, I21.x, I46.x) in the Danish National Patient Registry and case status was validated by reviewing hospital records [18] according to the current recommendations of the American Heart Association [19]. Within the cohort we defined *a priori* a sex stratified, random cohort sample (a sub-cohort) consisting of 999 men and 870 women randomly selected among all study participants.

### Genotyping

DNA was extracted from thawed lymphocytes from buffy coat preparations of EDTA-blood (5 mL tubes) using the salting-out method as described by Miller [20]. On average the yield was 100 ug from 1 ml buffy coat preparation. DNA concentration was estimated on basis of measurements of selected samples. Salting out gives very high quality high molecular weight DNA and purity was evaluated by measuring A260/A280.A260/A280 was 2.0 in TE buffer indicating very low protein contamination.

Inspired by study results showing an association with rupture of collagen-containing membranes in Caucasians when comparing the homozygous wildtype genotype with the homozygous variant genotype [15], two SNPs in the vitamin C transporter gene *SLC23A2*, rs6139591 and rs1776964, were selected for the present study. Rs6139591 and rs1776964 tag 26 and 3 other neighbouring SNPs, respectively, within the gene (r^2^>0.8, SNAP Database).

Cases and controls were mixed before genotyping and the lab personal were blinded to the case/control status during genotyping. Genotyping was performed using pre-designed TaqMan® genotyping assays (Applied Biosystems). Controls with known genotype and no template controls were included in each run. Genotyping was successful in 99% of the samples.

### Intake of dietary vitamin C

All cohort members completed a validated detailed 192-item food frequency questionnaire of which 44 of the items exclusively concerned intake of fruit, vegetables, or fruit/vegetables juice [21]. Participants were asked how often they consumed each food item on average over the past year, with 12 possible choices ranging from “never or less than once per month” to “8 times per day.” Total Vitamin C intake from food only was estimated by multiplying the frequency of consumed food by the nutrient content of the specified portion [22].

### Statistics

We used a case-cohort design with a sub-cohort of 1,869 subjects drawn randomly, stratified on sex from the whole cohort with 57,053 subjects. Genotype distributions were checked among the sub-cohort to verify Hardy Weinberg equilibrium using chi-squared test.

The observation time for each participant was the period from enrolment into the cohort until date of registered ACS event, death from other causes, emigration, loss to follow up or December 31, 2003 whichever came first [23]. Age was used as the time scale in the Cox regression model. Due to the *a priori* known difference in incidence rates of ACS between the two sexes, we employed sex-specific Cox proportional hazards models giving estimates of hazard ratios (HRs) and 95% confidence intervals (CI) to test the main genetic effects and the gene×diet interactions on acute coronary syndrome incidence. The intake of estimated daily intake of vitamin C, fruit, and vegetables was used as a binary exposure variable, such that these intakes were dichotomized at above or below their sex-specific median intake among cases. For each sex, we performed crude analyses and analyses adjusted for baseline values of established risk factors for ACS, i.e. BMI, LDL concentrations, systolic blood pressure, smoking status, physical activity, alcohol consumption, supplement intake, total energy intake, saturated fat intake, and fibre intake. Smoking status, physical activity and supplement intake were used as stratifying variables and the other covariates were added as categorical variables according to sex-specific quintiles. Time in study was included as a time-varying binary covariate, allowing the hazard ratio to change after one year in the study. Finally, we tested whether there was evidence of an interaction effect between SNPs and sex by including an interaction-term (SNP×sex) in the multivariable models not stratified by sex.

All models were modified by a weighting scheme as if the full cohort was included [24], and using a robust variance estimate. In the case-cohort design, weights were assigned to each subject, one for cases and N/n for non-cases in the sub-cohort, where N (n) is the number of non-cases in the cohort (sub-cohort). For women, N/n = 29,019/851 and for men, N/n = 25,142/929. We observed no serious violations against the proportional hazards assumption. Analyses were done using Stata version 11.2 (Stata Corporation, College Station, Texas, US). A value of *P*<0.05 (two-tailed) was taken to indicate statistical significance.

## Results

Participants were excluded from the analyses if information on one or more covariates was missing, or if genotyping failed. Thus, in this study, 936 cases and a sub-cohort of size 1,580 were included. The prevalence and distribution of clinical and lifestyle factors in cases and sub-cohort members are shown in Table 1. As expected, cases had increased occurrence of several known cardiovascular risk factors compared with the sub-cohort members. The median dietary intakes of vitamin C, vegetables, and fruits were significantly lower among cases than among sub-cohort members. Among cases, 86% were diagnosed with myocardial infarction, 12% with unstable angina pectoris and 2% with cardiac arrest.

**Table 1:** Baseline characteristics in the study population^1^.

Characteristics	Sub-cohort		Cases	
	Women	Men	Women	Men
	(n = 748)	(n = 832)	(n = 226)	(n = 710)
Age, y	55.8 (50.7–63.7)^2^	56.0 (50.8–64.2)	59.7 (51.7–64.7)	58.2 (51.1–64.7)
BMI, kg/m^2^	24.6 (19.8–33.6)	26.3 (21.5–32.3)	26.1 (20.0–35.3)	26.8 (22.4–34.1)
LDL, mmol/L	3.5 (2.1–5.2)	3.56 (2.38–5.07)	3.95 (2.35–5.78)	3.96 (2.49–5.40)
HDL, mmol/L	1.8 (1.2–2.6)	1.41 (1.02–2.12)	1.56 (1.07–2.27)	1.30 (0.97–1.91)
Systolic blood pressure, mmHg	149 (117–184)	140 (112–178)	151 (114–186)	149 (117–184)
Diastolic blood pressure, mmHg	87 (72–108)	84 (69–104)	86 (69–104)	87 (72–108)
Smoking status, % (n)				
Never	42% (317)	34% (541)	24% (54)	14% (102)
Former	22% (162)	29% (453)	16% (36)	28% (197)
Current	36% (269)	37% (586)	60% (136)	58% (411)
Use of supplements, % (n)	79% (594)	65% (537)	75% (170)	59% (418)
Vegetables, g/day	174 (51–367)	156 (48–363)	144 (48–367)	132 (38–328)
Fruits, g/day	201 (33–583)	144 (22–518)	188 (31–547)	124 (17–454)
Vitamin C from diet, mg/d	103 (45–231)	95 (42–203)	98 (44–213)	90 (37–182)
rs6139591				
CC	32.5% (243)	33.2% (276)	28.3% (64)	32.3% (229)
CT	49.5% (370)	47.6% (396)	49.6% (112)	48.5% (344)
TT	18.1% (135)	19.2% (160)	22.1% (50)	19.3% (137)
rs1776964				
CC	29.8% (223)	30.1% (250)	27.0% (61)	29.3% (208)
CT	47.3% (354)	49.8% (414)	52.7% (119)	48.6% (345)
TT	22.9% (171)	20.2% (168)	20.4% (46)	22.1% (157)

1 Only subjects with complete data are included.

2 Data are expressed as median (5^th^ and 95^th^ percentiles) or % (number) of subjects within the specific category.

DNA was available and genotypes of rs6139591 and rs1776964 were successfully determined for 2,671 and 2,678 participants, respectively. Both rs6139591 and rs1776964 were in Hardy–Weinberg equilibrium in the sub-cohort (p>0.05). The minor allele frequencies were 43.5% for the rs6139591 *T* allele and 45.9% for the rs1776964 *T* allele, respectively. The two single nucleotide polymorphisms were not in LD as tested by chi-square test (p>0.05).

Among women, rs6139591 was associated with risk of ACS. Compared with CC-carriers, the adjusted HR for heterozygotes was 1.64 (95% CI: 0.91–2.96) and 2.53 (1.23–5.20) for TT-homozygotes. In men, we found the same trend of a higher adjusted risk for rs6139591T-allele carriers although much weaker and not statistically significant (**Table 2**). We observed weaker, statistically non-significant associations between rs1776964 and ACS in both women and men (Table 2).

**Table 2:** Hazard ratio of acute coronary syndrome according to *SLC23A2* genotype in the Danish Diet, Cancer and Health Cohort.

	Crude Model	Adjusted Model^1^
*SLC23A2*	Hazard Ratio	Hazard Ratio
	(95%CI)	(95%CI)
**Women**		
*rs6139591*		
CC (n cases = 64)	1	1
CT (n cases = 112)	1.21 (0.84–1.74)	1.64 (0.91–2.96)
TT (n cases = 50)	1.48 (0.95–2.31)	2.53 (1.23–5.20)
*rs1776964*		
CC (n cases = 61)	1	1
CT (n cases = 119)	1.24 (0.86–1.78)	1.81 (0.99–3.32)
TT (n cases = 46)	1.08 (0.69–1.69)	1.79 (0.87–3.70)
**Men**		
*rs6139591*		
CC (n cases = 229)	1	1
CT (n cases = 344)	1.03 (0.82–1.30)	1.11 (0.80–1.54)
TT (n cases = 137)	1.00 (0.74–1.34)	1.25 (0.85–1.85)
*rs1776964*		
CC (n cases = 208)	1	1
CT (n cases = 345)	0.92 (0.73–1.17)	0.90 (0.65–1.24)
TT (n cases = 157)	1.07 (0.80–1.43)	1.25 (0.83–1.86)

1 Cox regression model adjusted for BMI, LDL concentrations, systolic blood pressure, smoking status, physical activity, alcohol consumption, supplement intake, total energy intake, saturated fat intake, fibre intake, and time in study.

Dietary vitamin C intake was dichotomized into high and low intake (sex-specific median intake among cases; women, 127 milligrams per day; men, 107 milligrams per day) and we observed that the association with rs6139591 was most consistent and most pronounced among women with lower than median intake.

Women with the TT genotype had a further detrimental effect of a low dietary vitamin C intake on ACS (adjusted HR, 5.39, 95% CI, 2.01–14.50) compared with CC-homozygotes. For heterozygotes, the risk was intermediate (adjusted HR, 1.56, 95%CI, 0.68–3.58) (**Table 3**). Among women with a high intake of vitamin C, only heterozygotes had a significantly increased risk (adjusted HR, 2.45, 95% CI, 1.04–5.79) compared with women being TT-carriers and having a low intake of vitamin C. Women carrying the rs1776964 TT-genotype had an increased risk of ACS when having a high intake of vitamin C compared with CC-homozygous women with low intake (adjusted HR, 3.42, 95% CI, 1.11–10.51).

**Table 3:** Hazard ratio of acute coronary syndrome according to *SLC23A2* genotype and dietary vitamin C intake.

		Crude	Crude	Adjusted^1^	Adjusted^1^
*SLC23A2*	N	Hazard Ratio	Hazard Ratio	Hazard Ratio	Hazard Ratio
		(95% CI)	(95% CI)	(95% CI)	(95% CI)
	Cases^2^	Dietary Vitamin C intake			
**Women**		≤127 mg/day^3^	>127 mg/day	≤127 mg/day	>127 mg/day
*rs6139591*					
CC	30/34	1.0	0.98 (0.55–1.74)	1.0	1.53 (0.52–4.55)
CT	57/55	1.30 (0.77–2.21)	1.11 (0.66–1.87)	1.56 (0.68–3.58)	2.45 (1.04–5.79)
TT	26/24	1.95 (1.02–3.71)	1.16 (0.62–2.19)	5.39 (2.01–14.50)	1.85 (0.62–5.51)
*rs1776964*					
CC	28/33	1.0	0.91 (0.51–1.65)	1.0	1.06 (0.37–3.07)
CT	67/52	1.52 (0.90–2.59)	0.91 (0.53–1.56)	1.99 (0.84–4.74)	1.66 (0.66–4.19)
TT	18/28	0.76 (0.38–1.52)	1.34 (0.70–2.44)	0.93 (0.30–2.93)	3.45 (1.16–10.28)
**Men**		≤107 mg/day^3^	>107 mg/day	≤107 mg/day	>107 mg/day
*rs6139591*					
CC	114/115	1.0	0.75 (0.52–1.07)	1.0	0.77 (0.46–1.31)
CT	169/175	0.88 (0.63–1.23)	0.89 (0.64–1.24)	0.93 (0.57–1.50)	1.00 (0.61–1.65)
TT	72/65	1.03 (0.68–1.58)	0.71 (0.47–1.09)	1.18 (0.66–2.11)	1.01 (0.57–1.79)
*rs1776964*					
CC	97/111	1.0	1.06 (0.72–1.55)	1.0	1.27 (0.73–2.20)
CT	181/164	1.15 (0.81–1.62)	0.80 (0.57–1.13)	1.26 (0.79–2.03)	0.81 (0.49–1.37)
TT	77/80	1.09 (0.72–1.67)	1.10 (0.73–1.67)	1.15 (0.64–2.08)	1.72 (0.95–3.11)

1 Cox regression model adjusted for BMI, LDL concentrations, systolic blood pressure, smoking status, physical activity, alcohol consumption, supplement intake, total energy intake, saturated fat intake, fibre intake, and time in study.

2 Numbers are representing subjects with low intake/high intake, respectively

3 Sex-specific median intake for cases.

As fruit and vegetables are the major food sources of dietary vitamin C, we also dichotomized the intake of vegetables and fruit into high and low intake (vegetables: sex-specific median intake among cases; women, 145 grams per day; men, 132 grams per day; fruit: sex-specific median intake among cases; women, 189 grams per day; men, 125 grams per day) and found the same indication of associations among women with a low intake as observed for dietary vitamin C intake (**Supplementary Table S1**, displaying for fruit). For women with a higher than median intake of vegetables or fruit, we observed a higher risk of ACS in homozygous subjects carrying the rs1776964 TT genotype compared to the CC homozygous women with a low intake. In men, we found no clear modifying effect of diet on the association between rs6139591 or rs1776964 polymorphisms and ACS.

## Discussion

In this prospective study, we examined the association between two common genetic variants in the sodium-dependent vitamin C transporter 2 and risk of acute coronary syndrome. We show that the polymorphism rs6139591 in *SLC23A2* was associated with ACS in women. We further demonstrated that the association was stronger in women with a low intake of dietary vitamin C, or with low intake of the primary sources of dietary vitamin C namely vegetables or fruit; for instance, TT homozygous women with dietary vitamin C intake <127 milligrams per day had a five-fold higher risk of acute coronary syndrome compared to CC homozygous women with the same vitamin C intake. Adjustment for known biological and lifestyle cardiovascular risk factors; i.e. age, BMI, LDL concentrations, systolic blood pressure, smoking status, physical activity, alcohol consumption, supplement intake, total energy intake, saturated fat intake, and fibre intake only strengthened the observed associations. The results support a role for variations in the *SLC23A2* gene in relation to risk of acute coronary syndrome in women with a low intake of dietary vitamin C. However, we also observed that for women with a high intake of dietary sources of vitamin C another common variant in the same gene was associated with a higher risk of ACS. Hence, the study suggests that the genotype effects may not be fully compensated by a higher intake of dietary vitamin C.

Several gene variants have been related to cardiovascular risk or risk factors [25], [26]. However, this may be the first time that genetic variation in the vitamin C transporter gene *SLC23A2* is tested in relation to cardiovascular disease, hence the results require replication. In support of our findings, a few other studies have confirmed that genetic variation in *SLC23A2* is associated with risk of adverse effects in other settings where vitamin C has been suggested to play a role, either due to its anti-oxidative properties and/or its function as cofactor in the synthesis of collagen [15], [27]. However, only for rs6139591 the direction of the association was comparable with the results of Erichsen *et al's* study on risk of preterm birth due to premature ruptures of membranes [15], which seems specifically related to vitamin C intake [28] reinforcing the necessity of replication.

SVCT2 is responsible for the bioaccumulation of vitamin C in contrast to SCVT1 which is responsible for dietary absorption and renal re-absorption. Animal studies have demonstrated that in knockout mice (slc23a2 −/−) with a complete deficiency of SVCT2 death is almost instant after birth, while the heterozygous mice (slc23a2 +/−) survive into adulthood but have low tissue accumulation of vitamin C [10]. In a mouse model that overexpresses additional copies of SVCT2, increased vitamin C levels were demonstrated in several tissues and an improved protection against oxidative stress was observed in lung tissue [29]. However, only a few studies have examined the functional consequences of the genetic variation in humans but it is likely to affect RNA transcription, stability and translation, or post-translational processes [16]. Hence, although the possible functional relevance of the studied genetic variants to ACS is not clear, it is possible that they disturb the intracellular uptake of vitamin C. It has been demonstrated that binding and bridging of several transcription factors within the promoter region control *SLC23A2* expression [30]. Hence, these two polymorphisms may result in impaired substrate-dependent regulation of the transporter gene expression [31]. Alternatively, the two polymorphisms considered here are not the causal variants *per se*, but rather in linkage disequilibrium with one or more risk modifying variations in *SLC23A2*. Whatever the mechanism, the variants may contribute to a disturbed intracellular uptake and hence a decreased intracellular ascorbate concentration. In a minor subsample of the DCC participants, a correlation between increased plasma vitamin C concentrations and increased dietary vitamin C intake has been demonstrated [32]. However, this relationship is non-linear as the plasma concentrations increase disproportionally with increasing intake suggesting a saturable or a ceiling effect [33] of plasma concentrations. Unfortunately, the present study is limited by the lack of measurements of intracellular ascorbate concentration as biological material in the study was not collected with that purpose. Hence, it is not possible to predict the influence on intracellular levels caused by potential defects in SVCT2 [16].

We observed the strong association only in women. It is not unusual that sex-specific relationships exist between ACS and various risk factors including genetic variants [34]–[36]. A common suggestion to explain this conundrum is that the expression of the gene is regulated by sex hormones [34]. However there have been no previous demonstrations that this is the case for *SLC23A2*. Furthermore, although the effect was much stronger and statistically significant in women compared with men, we did not observe interaction between genotype and sex in a formal test. Thus, as the study is very likely underpowered to detect interaction and since women constitutes a much smaller case group, these results call for replication in future studies.

Strengths of the present study include the almost complete follow-up, which may reduce selection bias, that information on dietary intake was obtained before diagnosis of ACS [18], which ensured collection of baseline exposure information independent of future outcome, and the fact that all cases were validated by review of medical records.

It is a limitation of the study that the participants (<34% of the invited subjects) are likely to differ from the general population. However, it is not expected that the association between the genotype and ACS will be different comparing participants with non-participants. Those who chose to participate may have a tendency to over-report the intake of fruit and vegetables and hence, the estimated intake of dietary vitamin C. However, we do not expect this over-reporting to be different between cases and controls. Finally, residual confounding cannot be excluded, however, we adjusted for a range of potential confounders in the various models and the associations only became stronger. Thus, confounding seems an unlikely explanation for the study results.

In summary, genetic variation in the sodium-dependent vitamin C transporter-2 gene were strongly associated with risk of acute coronary syndrome in women. Taking into account the frequency of the detrimental genotype, the results may be of public health concern.

## Supporting Information

Table S1 **Hazard ratio (HR) of acute coronary syndrome according to** ***SLC23A2*** **genotype and fruit intake.**
(DOCX)
