# Supplementary material for: Variation in the Sodium-Dependent Vitamin C Transporter 2 Gene Is Associated with Risk of Acute Coronary Syndrome among Women
Source: PLoS One. 2013 Aug 21;8(8):e70421. doi: 10.1371/journal.pone.0070421 (PMC3749152; doi:10.1371/journal.pone.0070421)
Supplement: Table S1 — Hazard ratio (HR) of acute coronary syndrome according to SLC23A2 genotype and fruit intake. (DOCX) [file pone.0070421.s001.docx]

**Table S1.** Hazard ratio (HR) of acute coronary syndrome according to *SLC23A2* genotype and fruit intake

|  |  | **Crude** | **Crude** | **Adjusted^1^** | **Adjusted^1^** |
| --- | --- | --- | --- | --- | --- |
| ***SLC23A2*** | **N** | **Hazard Ratio** | **Hazard Ratio** | **Hazard Ratio** | **Hazard Ratio** |
|  |  | **(95% CI)** | **(95% CI)** | **(95% CI)** | **(95% CI)** |
|  | **Cases^2^** | **Fruit intake** | | | |
| **Women** |  | ≤ 189 g/day**^3^** | > 189 g/day | ≤ 189 g/day | > 189 g/day |
| *rs6139591* |  |  |  |  |  |
| CC | 35 / 29 | 1.0 | 0.71 (0.40 - 1.25) | 1.0 | 0.72 (0.26 - 1.99) |
| CT | 52 / 60 | 0.99 (0.59 - 1.65) | 1.05 (0.63 - 1.73) | 0.85 (0.39 - 1.87) | 2.54 (1.15 - 5.62) |
| TT | 26 / 24 | 1.62 (0.87 - 3.04) | 1.00 (0.54 - 1.86) | 3.14 (1.22 - 8.05) | 1.64 (0.57 - 4.76) |
| *rs1776964* |  |  |  |  |  |
| CC | 28 / 33 | 1.0 | 0.96 (0.54 – 1.74) | 1.0 | 1.54 (0.56 – 4.28) |
| CT | 65 / 54 | 1.44 (0.85 – 2.45) | 1.01 (0.59 – 1.73) | 2.10 (0.88 – 5.03) | 2.41 (0.96 – 6.06) |
| TT | 20 / 26 | 1.97 (0.50 – 1.89) | 1.13 (0.60 – 2.16) | 1.46 (0.51 – 4.23) | 3.42 (1.11 – 10.51) |
| **Men** |  | ≤ 125 g/day**^3^** | > 125 g/day | ≤ 125 g/day | > 125 g/day |
| *rs6139591* |  |  |  |  |  |
| CC | 114 / 115 | 1.0 | 0.60 (0.42 – 0.87) | 1.0 | 0.73 (0.42 – 1.26) |
| CT | 171 / 173 | 0.83 (0.58 – 1.17) | 0.73 (0.52 – 1.03) | 0.88 (0.53 – 1.44) | 0.99 (0.58 – 1.66) |
| TT | 70 / 67 | 1.06 (0.68 – 1.66) | 0.57 (0.38 – 0.87) | 1.41 (0.77 – 2.58) | 0.84 (0.47 – 1.50) |
| *rs1776964* |  |  |  |  |  |
| CC | 91 / 117 | 1.0 | 0.87 (0.60 – 1.28) | 1.0 | 1.28 (0.75 – 2.20) |
| CT | 187 / 158 | 1.10 (0.77 – 1.58) | 0.67 (0.47 – 0.96) | 1.25 (0.78 – 2.02) | 0.81 (0.48 – 1.38) |
| TT | 77 / 80 | 1.15 (0.74 – 1.79) | 0.86 (0.57 – 1.31) | 1.44 (0.78 – 2.64) | 1.40 (0.77 – 2.54) |

^1^ Cox regression model adjusted for BMI, LDL concentrations, systolic blood pressure, smoking status, physical activity, alcohol consumption, supplement intake, total energy intake, saturated fat intake, fibre intake, and time in study.

^2^ Numbers are representing subjects with low intake / high intake, respectively

**^3^** Sex-specific median intake for cases.
